# Vitreoretinal interface abnormalities in patients treatedwith ranibizumab for diabetic macular oedema

**DOI:** 10.1007/s00417-016-3562-0

**Published:** 2016-12-12

**Authors:** Yun Wong, David H. W. Steel, Maged S. Habib, Alex Stubbing-Moore, Dalvir Bajwa, Peter J. Avery, The Sunderland Eye Infirmary study group, Maged S. Habib, Ajay Kotagiri, Maria T. Sandinha, Jonathan M. Smith, David H. W. Steel, Deepali Varma

**Affiliations:** 1 Sunderland Eye Infirmary, Queen Alexandra Road, Sunderland, SR2 9HP UK; 2 Institute of Genetic Medicine, Newcastle University, Newcastle Upon Tyne, UK; 3 School of Mathematics & Statistics, Newcastle University, Newcastle Upon Tyne, UK

**Keywords:** Anti VEGF, Ranibizumab, Epiretinal membrane, Vitreomacular adhesion, Diabetic macular oedema, Vitreoretinal interface abnormality, Real-world outcomes

## Abstract

**Purpose:**

Intravitreal anti-vascular endothelial growth factor (VEGF) agents are effective in the treatment of central involving diabetic macular oedema (DMO). Vitreoretinal interface abnormalities (VRIA) are common in patients with DMO, and the effect of these on the response to anti-VEGF treatment is unclear. Furthermore the effect of anti-VEGF agents on the VRIA itself is uncertain.

**Method:**

Prospective study of consecutive patients treated with ranibizumab (RZB) for DMO as part of routine clinical care in one eye unit over a 1-year period. Visual acuity (Va), central retinal thickness (CRT) and injection frequency data was recorded on an electronic database. Treatment was initiated with four monthly RZB injections and then a monthly PRN regime. All patients underwent high-density spectral-domain optical coherence tomography (SDOCT) at baseline and 12 months. The SDOCTs were graded by two observers masked to the outcome.

**Results:**

One hundred and four eyes (77 patients) were included in the analysis. The mean age was 62 years, and 62% were male. The mean presenting vision was 62 letters and CRT 472 μm. Eighty eyes retained stable Va, and 17 had an improvement in Va. At baseline, 39 eyes had associated focal vitreomacular adhesion (VMA) and by 12 months this reduced to 30 (*p* = 0.04), with 12 releasing VMA and three developing it. Patients with VMA had significantly better final Va than those without VMA. Improvement in CRT was greatest in those where VMA released during the study. Forty-five eyes had some degree of foveal involving epiretinal membrane (ERM) at baseline, and 28 were considered to have clinically significant ERM. There was no clinically relevant change in ERM during the study. Patients with significant ERM at baseline had a lower final vision. Multivariate analysis showed that ERM and more severe retinopathy at baseline were predictive of less visual improvement (*p* < 0.01). Shorter intraretinal cyst length, ERM and the absence of VMA at baseline were predictive of a worsened anatomical response (*p* < 0.001).

**Conclusion:**

VRIA are related to outcome in patients treated with RZB. ERM was associated with a worsened visual and anatomic response, and VMA with an improved anatomical response particularly when spontaneous VMA release occurred during treatment. The presence and severity of ERM was not affected by RZB treatment.

**Electronic supplementary material:**

The online version of this article (doi:10.1007/s00417-016-3562-0) contains supplementary material, which is available to authorized users.

## Introduction

It is well known that there is a high prevalence of vitreoretinal interface abnormalities (VRIA) in patients with diabetic macular oedema (DMO [1–6]. Epiretinal membrane (ERM) and incomplete vitreoretinal separation with vitreomacular attachment (VMA) and traction (VMT) have been described and related to pathological changes in the vitreous and vitreoretinal interface [7–9]. As well as the association of VRIA with DMO, a causative role has also been postulated and surgical relief of traction demonstrated to be of benefit in some patients [10–12].

Anti-VEGF agents have been shown to improve clinical outcomes in patients with centre involving DMO compared to laser [13]. The presence, however, of VRIA on the response to anti-VEGF agents in patients with DMO has had limited study, although there is some data to suggest that they reduce the therapeutic effect [14]. These agents have also been shown to alter the balance between angiogenic and fibrotic growth factors in patients with diabetic retinopathy, termed the angiofibrotic switch which can result in increased retinal traction in some patients with proliferative diabetic retinopathy (PDR) prior to surgery [15].

We carried out a prospective study on a consecutive cohort of patients undergoing treatment with ranibizumab (RZB) for centre involving DMO to evaluate the effect of treatment on the VRIA and also to assess whether the presence of VRIA had any effect on treatment outcomes.

## Method

Information on consecutive patients commenced on RZB for DMO between May 2013 and May 2014 at one ophthalmic treatment centre (Sunderland Eye Infirmary, UK) were prospectively entered onto an electronic data collection form. Patients were eligible for treatment as per UK National Institute for Health and Care Excellence criteria with centrally involving DMO with a foveal retinal thickness of greater than 400 μm. Visual acuity (Va) was measured using an Early Treatment Diabetic Retinopathy Study (ETDRS) chart at 2 m with best correction. Patients underwent spectral-domain optical coherence tomography (30 by 30° horizontal grid protocol with 60 μm line spacing) using a Spectralis HRA + SDOCT (Heidelberg Engineering, Heidelberg, Germany) at baseline and 12 months. Treatment was initiated with four monthly RZB injections, with an additional two following this if the oedema had not resolved (central retinal thickness (CRT) <250 μm) or vision was less than 85 letters followed by further injections as necessary using the Diabetic Retinopathy Clinical Research Network protocol I and monthly follow-up [13]. At 12-month follow-up, SDOCT was obtained using the Spectralis AutoRescan feature, matching the position of the 12-month scan to the exact position of the baseline scan.

At baseline the following information was recorded: age, sex, type and duration of diabetes, HBA1c, retinopathy grade (background, pre proliferative, active proliferative or inactive treated proliferative retinopathy), and the occurrence of previous focal laser with the dates of the first macular laser as a surrogate for DMO duration. At each visit, Va, CRT, and whether an injection or laser was given was recoded, but for this study only the baseline and 12-month data were used.

The baseline and 12-month SDOCTs were graded by an observer masked to the outcome. The following parameters were recorded: central retinal thickness (CRT: average retinal thickness over the central 1 mm^2^), maximum retinal thickness (MRT) anywhere in the central 1 mm^2^, the presence of subretinal fluid, the presence of intraretinal cysts and the vertical length of the longest cysts within the central 1 mm^2^ if present, and the integrity of the ellipsoid zone graded as intact, focally disrupted, or more severely disrupted based on a gap of >1,000 μm in the central 1 mm^2^.

A variety of measures of the vitreoretinal interface were recorded and graded by two observers, with the final grade made by consensus in cases of disagreement. These included: the presence of VMA (defined as perifoveal vitreoretinal separation within 2,500 μm of the foveal centre in any direction with persistent vitreoretinal attachment at the fovea) and the longest horizontal width of the attached zone through the foveal centre if present, the presence of any foveal (within central 1 mm^2^) and eccentric (outside central 1 mm^2^ but within 5 mm ETDRS circle) ERM (defined as a hyper-reflective inner retinal band and graded as not present, present, or associated with retinal plication and/or peg-like attachments). Baseline ERM was designated as clinically significant if the following criteria were met:

1. ERM involving the foveal centre associated with a change in foveal architecture and/or retinal surface wrinkling on the fundal image.
2. Eccentric ERM if associated with retinal plication and/or retinal surface wrinkling and in continuity with the central zone of retinal thickening

The baseline and 12-month OCTs were compared to assess whether there had been a change in the ERM (i.e., new ERM, or the development of signs of retinal contracture in a linear ERM) or VMA.

We defined stability of vision as being within 10 letters of baseline Va, and improvement/reduction as being a change of greater than 10 letters. Anatomical response was defined as a reduction in CRT of 15% of baseline or more.

To be eligible for inclusion in the final analysis, patients had to have had follow-up for 12 months after first injection with baseline and 12-month SDOCT, and at least four consecutive RZB injections at the beginning. Patients with inactive PDR previously treated with panretinal photocoagulation and patients with previous focal laser injection for DMO were included, provided there had been no laser within 3 months of the first RZB .

Patients were excluded if they had visually significant cataract or cataract surgery during or within 3 months of the study period, active PDR at baseline requiring panretinal photocoagulation, other intravitreal agents during the study period, and previous vitrectomy surgery. Under local protocols, patients with significant tractional changes associated with incomplete vitreoretinal separation (i.e., focal vitreomacular attachment with alteration of foveal architecture) and DMO were treated with vitrectomy and were not included.

Under UK guidelines the analysis was classified as a service evaluation, and as such did not require ethical approval.

## Statistical analysis

Descriptive and statistical analysis was performed using Minitab 17 (Minitab Ltd, Coventry, UK). Patients’ demographic baseline characteristics are presented in terms of mean, standard deviation (SD), and range or percentage as appropriate. Similar information is presented when the data was divided into groups. *T*-tests and one-way ANOVA were used to compare continuous variables and chi-squared tests on the categorical variables. A log transformation was used to achieve normality if the Anderson–Darling statistics gave *p*-values < 0.05 on the untransformed data. Mann–Whitney U-tests were used on discrete data. When looking at the change in variables following treatment, matched pairs *t*-tests were used for continuous variables and McNemar’s test for binary variables. For categorical variables with three categories, three binary tables were produced and the minimum *p*-value for the three McNemar’s test reported. General linear modelling with a stepwise option was used to distinguish the most important variables and present a predictive model for Va and CRT change. Mixed modelling, fitting individual as a random effect, was used to check the significant results in cases where there was a high intra-class correlation.

## Results

During the study period, 132 eyes (96 patients) were treated with RZB for DMO. Twenty-eight eyes (19 patients) were excluded because of incomplete follow up (ten), cataract surgery (two), active PDR (eight) and previous vitrectomy surgery (three) as per our exclusion criteria, leaving 104 eyes (77 patients) for analysis.

## Baseline features and overall response rate

The baseline features of the patients are shown in Table 1. The mean patient age was 62 years (range 29–89), with 87% having type 2 diabetes. Out of 77 included patients, the mean % haemoglobin A1c was 8.2 and mean Va was 62 letters. The mean CRT was 472, and 80% of the patients had pre-proliferative or treated proliferative retinopathy. Sixty-six percent of patients had previously been treated for maculopathy with laser. Eleven of the cohorts were current smokers. Eighteen patients (23%) had a history of ischaemic heart disease, and 13(17%) of cerebral vascular disease. None of the patients in this cohort were receiving dialysis during the study period or had had a renal transplant.

**Table 1:** Demographics of entire cohort

Age: mean, SD (range)	62.1, 13.0 (29–89)
Sex *n* (%) (female/male)	29 F/48 M (38% / 62%)
Type of diabetes *n* (%) Type 1/Type 2	10/67 (13%/ 87%)
Diabetes duration: mean, SD (range)	14.3, 8.6 (1–45)
% Haemoglobin A1c: mean, SD, (range)	8.2, 1.7 (5–13.7)
Baseline Va: mean, SD (range)	61.6, 15.6 (5–85)
Baseline CRT: mean, SD (range)	471.8, 113.1 (270–856)
Baseline MRT: mean, SD (range)	544, 108 (400–968)
Retinopathy grade in treated eye *n* (%) for BDR, PPDR, PDR)	21, 43, 40 (20%, 41%, 39%)
Previous macular laser in treated eye *n* (%)	69 (66%)
Days to first laser for maculopathy: mean, SD (range)	660, 532 (91–1910)
Height of longest intraretinal cyst in microns: mean, SD (range)	295, 152, (0–947)
Presence of outer retinal changes *n* (%) for none, mild, severe	(32, 30, 42) (31%, 29%, 40%)
Presence of SRF *n* (%)	40 (38%)

*BDR* background diabetic retinopathy, *PPDR* pre proliferative retinopathy, *PDR* inactive treated proliferative retinopathy, *CRT* central retinal thickness, *SD* standard deviation, *SRF* subretinal fluid

The overall 12-month outcomes for the cohort are given in Table 2. The mean number of injections was seven (range 4–11). Seventy-seven percent retained stable vision, whilst 16% had an improvement in vision. The mean change in visual acuity was +3.4 letters and in CRT −127 μm. Overall, 73% had an anatomical response to RZB.

**Table 2:** Outcomes at 12 months for entire cohort

Number of injections	
Mean, SD (range)	6.75, 1.51 (4–11)
Visual acuity stable *n*, %	80, 77%
Visual acuity worse *n*, %	7, 7%
Visual acuity improved *n*, %	17, 16%
Visual acuity change	
Mean, SD (range)	+3.4, 9.3 (−28–38)
CRT change	
Mean, SD (range)	−127, 116 (−422–203)
Anatomical response *n*, %	76, 73%

*CRT* central retinal thickness, *SD* standard deviation

Changes in SD OCT features of the cohort are shown in Table 3. Both Va and retinal thickness decreased significantly.

**Table 3:** Changes in SD-OCT features pre and post treatment in treated eyes

	Baseline	Post	*P*
Va mean, SD (range)	61.6, 15.6 (5–85)	65.0, 13.9 (26–87)	<0.001^a^
CRT, mean, SD (range)	472, 113 (270–856)	345,100 (203–758)	<0.001^a^
MRT mean, SD (range)	544, 108 (400–968)	410, 103 (274–813)	<0.001^a^

*CRT* central retinal thickness, *MRT* maximum retinal thickness, *SD* standard deviation, *Va* visual acuity, *SD OCT* spectral-domain optical coherence tomography

^a^matched pairs *t*-test

## Vitreomacular adhesion

At baseline, 39 eyes (37.5%) had associated VMA. At 12 months this had reduced to 30 (28.8%) (*p* = 0.04), with 12 eyes releasing VMA and three developing it. The mean VMA width at baseline was 3,149 μm, which reduced to 2,732 μm post-treatment. There was a weak relationship between the VMA width at baseline and its subsequent release, with those releasing having a mean width of 2,418 μm (range 242–2,479 μm) (*p* = 0.06).

The baseline features and outcome of the patients with and without VMA at baseline are shown in Table 4. There were more male patients with VMA than female. Patients with VMA had both better presenting and final Va than those without VMA. However, only the final Va remained significant (*p* = 0.05) when mixed modelling was used. Otherwise there were no significant differences.

**Table 4:** Comparison between those with and without VMA at baseline

	VMA at baseline, *n* = 39	No VMA at baseline, *n* = 65	*P*
Age, years			
Mean, SD (range)	60.7, 9.5 (29–71)	63.0, 14.8 (30–89)	0.47
Sex, *n*	7 female, 22 male	22 female, 26 male	
(%) (female/male)	(24%/76%)	(46%/54%)	0.05
Retinopathy grade in treated eye: *n*, (% for BDR, PPDR, PDR)	10, 18, 11	11, 25, 29	
	(26%, 46%, 28%)	(16%, 38%, 45%)	0.23
Baseline visual acuity			
Mean, SD (range)	65.4, 12.3 (34–85)	59.3, 17.0 (5–85)	0.05
Final visual acuity			
Mean, SD (range)	69.2, 13.6 (31–87)	62.5, 13.6 (26–84)	0.02
Baseline CRT			0.47^a^
Mean, SD (range)	458, 86 (305–676)	480, 127 (270–856)	
Final CRT			
Mean, SD (range)	321, 81 (228–654)	359, 109 (203–758)	0.06^a^

*CRT* central retinal thickness, *MRT* maximum retinal thickness, *SD* standard deviation, *Va* Visual acuity, *BDR* background diabetic retinopathy, *PPDR* pre proliferative retinopathy, *PDR* inactive treated proliferative retinopathy

^a^log transformed

**Fig. 1:**
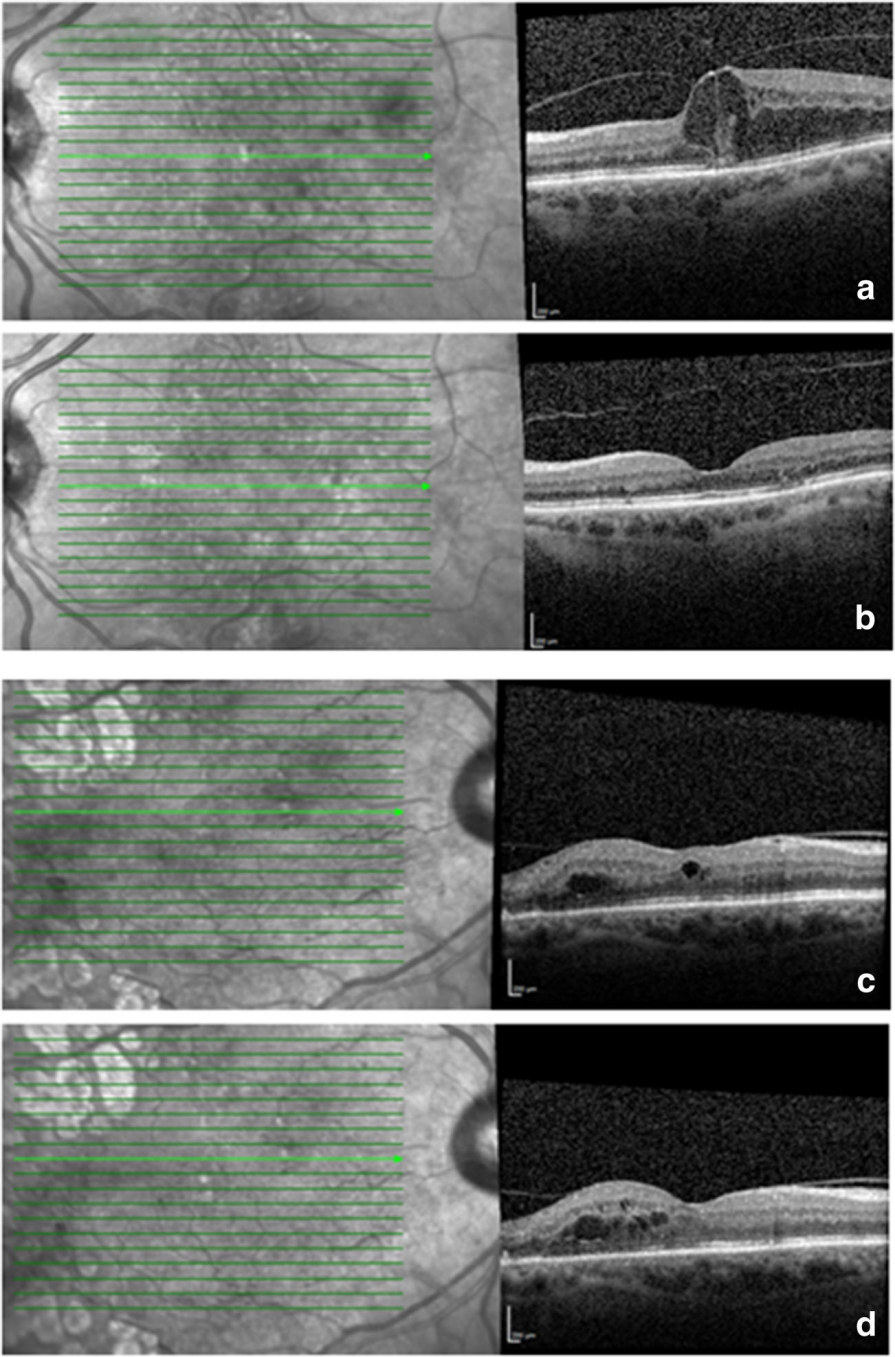
Two patients with focal (**a**) and broad (**c**) VMA at baseline. First patient (**a**) has spontaneous VMA release with good anatomical response at 12 months (**b**) whilst second patient (**c**) has persistent VMA and retinal thickening (**d**)

Table 5 divides the presence of VMA into four groups: VMA not present at any point in study, VMA present at baseline and 12 months, VMA present at baseline and released at 12 months, and finally those with VMA that developed during the study. There was a significant difference in the improvement in CRT, with the greatest improvement in those in whom VMA was present at baseline which then released during the study (Fig. 1).

**Table 5:** Comparison between the four VMA subgroups

	VMA group				
	No VMA (*n* = 62)	VMA during entire study period (*n* = 27)	VMA at baseline that released during study (*n* = 12)	VMA that formed during study (*n* = 3)	*P*
Change in Va, mean	3.3, 10.6	4.1. 7.0,	3.2, 8.4	−1.0, 1.0,	0.85
SD (range)	(−28 to 38)	(−15 to 17)	(−17 to 16)	(−2.0 to 1.0)	
CRT change, mean	−125, 122	−101, 103	−217, 80	−44, 60	0.01
SD (range)	(−422 to 203)	(−288 to 166)	(−339 to −49)	(−94 to 23)	
Injections; mean	6.7, 1.4	6.9, 1.6	6.5, 1.6	7.3, 3.2	0.85
SD (range)	(4–9)	(4–11)	(4–9)	(5–11)	

*VMA* vitreomacular adhesion, *CRT* central retinal thickness, *SD* standard deviation, *Va* visual acuity

## Epiretinal membrane

Forty-three percent of the cohort had some degree of foveal involving ERM at baseline, and 63% some eccentric ERM. In approximately one third of these patients, the ERM was associated with retinal contracture with either retinal plication or pegs visible. The total number of patients who were graded as having significant ERM at baseline was 28. No patient developed clinically significant ERM at 12 months who didn’t have it at baseline. There were 19 patients who developed a one-step progression in ERM (i.e., who either developed new ERM or who progressed from linear ERM to evidence of contracture) from baseline to 12 months, and 21 patients who had a reduction in ERM signs (no significant difference: see supplementary tables S1 and S2). There was no association between the presence of baseline ERM and its progression (*p* = 0.89)

**Fig. 2:**
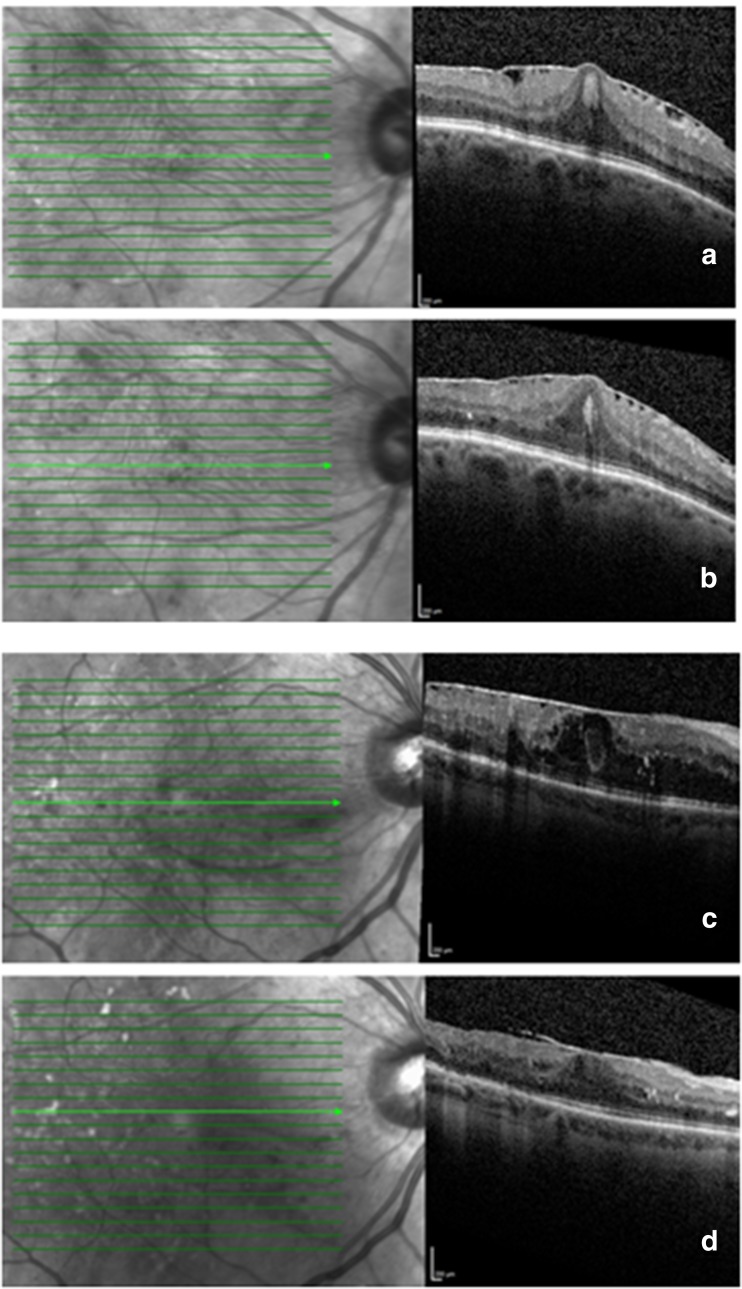
Two patients with foveal involving ERM at baseline (**a**, **c**) and at 12 months (**b**, **d**). Both have signs of ERM contracture with retinal plication which persist at 12 months. **d** shows some evidence of auto-release temporally but both show persistent thickening

Table 6 compares the features of those patients with and without clinically significant ERM at baseline and 12 months. There were significantly more female patients, more type 1 diabetics and more with previously treated proliferative diabetic retinopathy with significant ERM than without. Patients with clinically significant ERM at baseline had a lower starting visual acuity than those without, and significantly worse final visual acuity. As before, the difference in baseline visual acuity becomes non-significant when mixed modelling is used, but the difference in final visual acuity is still highly significant (*p* < 0.01). There was no difference in baseline CRT between the groups, but the final CRT was significantly worse in the ERM group (Fig. 2). The conclusions are the same if mixed modelling is used. There was no significant difference in the total number of injection received over the treatment period (*p* = 0.71), the duration of maculopathy (*p* = 0.75), or a history of previous laser (*p* = 0.79).

**Table 6:** Comparison between those with and without clinically significant ERM

	Significant ERM at baseline, *n* = 28	No significant ERM at baseline, *n* = 76	*p*
Age; mean, SD, (range)	58.1, 17.1 (29–83)	63.7, 10.7 (30–89)	0.09
Sex n (%) (female/male)	13 F, 9 M (59%/31%)	16 F,39 M (29%/71%)	0.01
Type of diabetes			
*N* (%) Type 1/Type 2	7/15 (32%/68%)	3/52 (5%/95%)	0.005
Retinopathy grade in treated eye: *n*	(2, 8, 18)	19, 35, 22)	0.003
(% for BDR, PPDR, PDR)	(7%, 29%, 64%)	(25%, 46%, 29%)	
Baseline visual acuity			
Mean, SD (range)	57.4, 15.8 (17–83)	63.2, 15.4 (5–85)	0.10
Final visual acuity			
Mean, SD, (range)	58.9, 17.2 (26–85)	67.3, 11.9 (38–87)	0.006
Baseline CRT			
Mean, SD (range)	486, 149 (324–856)	467, 97 (270–785)	0.70^a^
Final CRT			
Mean, SD (range)	407, 136 (219–758)	322, 72 (203–607)	<0.001^a^

*ERM* epiretinal membrane, *BDR* background diabetic retinopathy, *PPDR* pre proliferative retinopathy, *PDR* inactive treated proliferative retinopathy, *CRT* central retinal thickness, *SD* standard deviation, *Va* visual acuity

^a^log transformed

## Multivariate analysis of factors affecting change in outcomes with treatment

Multivariate analysis of the effect of all baseline features on visual acuity and CRT change showed that the presence of an increasing degree of ERM (clinically significant ERM > presence of foveal ERM with pegs or plication > linear foveal ERM) and more severe retinopathy (proliferative > pre-proliferative > background retinopathy) at baseline were predictive of less visual improvement (*R* ^2^ = 15%, *p* < 0.01).

Shorter intraretinal cyst length, the presence of increasing degrees of ERM, and the absence of VMA at baseline were predictive of a worsened anatomical response (*R* ^2^ = 42%, *p* < 0.001).

No other baseline features including previous focal laser and the duration of the DMO were predictive of outcome

## Discussion

We describe a significant influence of vitreoretinal interface abnormalities on anatomical and visual outcomes after intravitreal RZB in patients with DMO. The effect was greater than other differences previously described as being prognostically important, including the presence of SRF and age [16, 17].

We found a high prevalence of ERM at baseline in patients with DMO, in keeping with previous studies [1, 2], and found that the presence of ERM was predictive of a more limited response to RZB for both improvement in Va and reduction in CRT at 12 months.

Only a few studies have investigated the effect of ERM on response to anti-VEGF agents. Wu et al. studied 30 eyes treated with one anti-VEGF injection, and found an adverse effect on Va and CRT response [18]. Yoon et al. studied 15 eyes after three injections, and found a negative effect on both outcomes [19]. Importantly, however, both studies grouped all vitreoretinal interface abnormalities together, including ERM and VMA. Similarly Bressler et al. found an association of ‘surface wrinkling’ retinopathy on fundal photographs, with poorer visual outcome after RZB [17]. This was undefined and could have been related to vitreoretinal attachment. They did not find that ‘OCT vitreoretinal abnormalities’ were associated with Va outcome, but again this was a broad group and difficult to interpret.

There are a number of reasons why eyes with pre-operative ERM may have a more limited response to anti-VEGF agents. It may simply represent more severe baseline disease, with chronic structural changes limiting potential visual improvement. In support of this hypothesis, we found an association between the presence of ERM and previously treated PDR and poorer baseline Va. Against this, however, we found no association between our surrogate measure of chronicity of DMO and the occurrence of clinically significant ERM, nor the frequency of prior macular laser. Alternatively, the ERM may be limiting RZB penetration to the tissue and/or preventing the restoration of normal anatomy and function mechanically.

We observed a relatively high prevalence of vitreomacular adhesion at baseline similar to previous studies [20]. Recently, Sadiq et al. showed a positive association of VMA with visual acuity, but not anatomical outcome in patients treated with DMO in the READ-3 study participants [19]. At baseline and at 12 months the Va in our VMA group was better than the group without VMA concurring with Sadiq et al. However, on multivariate analysis we found that only the anatomical response to RZB was improved in the presence of VMA, with the greatest response seen in those with VMA at baseline who subsequently released the adhesion prior to the 12-month visit.

VMA release was relatively common, occurring in approximately 25% of our cohort with VMA at baseline, similarly to the findings of Sadiq et al. [19] This is in contrast to Sivaprasad et al., who found an infrequent occurrence of vitreomacular separation in patients with DMO after intravitreal triamcinolone [21], and the 10% release rate in the control arm of the MIVI Trust trial [22]. This perhaps relates to the number of intravitreal injections given, a known precipitant of vitreous separation. We only analysed SD OCT changes at 12 months, so do not know when VMA release occurred in our patients. We did, however, find that a narrower attachment of VMA was associated with a greater incidence of release during treatment. Sivaprasad et al. and Sadiq et al. both described an improvement in CRT after vitreoretinal release, as we found. This is in contrast to the generally low incidence of VMA release in wet AMD patients and the negative effect of VMA at baseline on response to anti-VEGF agents, albeit mainly on the need for and frequency of repeat injections [23–25]. We did not find a difference in the number of RZB injections on our PRN regime, but our study is limited in this regard by its real-world nature. The difference in the importance of VMA at baseline between wet AMD and DMO is difficult to explain, but our data support the findings of Sadiq et al. that in DMO patients it should not be interpreted as an indicator of a worsened prognosis.

There have been case reports of patients treated with DMO using anti-VEGF agents developing a variety of new VRIAs [26–28]. We, however, did not find any evidence for a systematic worsening in vitreoretinal traction in patients being treated for DMO. Overall, 19 patients showed worsening of ERM, but 21 patients showed signs of improvement in ERM and no patient developed traction to an extent to warrant vitrectomy. This is contrary to the experience in patients with active PDR being treated with anti-VEGF agents prior to vitrectomy, where increased retinal traction has frequently been observed [29, 30]. It is thought that it is the alteration in the balance between connective tissue growth factor (CTGF) and VEGF that results in increasing fibrosis after anti-VEGF use, termed the angiofibrotic switch [15]. Vitreous VEGF levels are known to be higher in PDR [31, 32] than in patients with DMO and NPDR, and CTGF levels are known to correlate with the degree of intraocular fibrosis. Indeed, we found a positive association between treated PDR and the occurrence of clinically significant ERM in our cohort. However, we did not find any association between the presence of ERM at baseline and any worsening of ERM during treatment.

We excluded patients with active PDR requiring further pan retinal photocoagulation, and it may be that these patients with DMO would have a higher risk of VRIA exacerbation with anti-VEGF agents.

Comparing our data to the RZB and deferred laser arm in the landmark DRCR net trial [13], our overall visual results are considerably worse (Va gain +3.4 in our study compared to +9 in the DRCR net), although interestingly the anatomical responses were similar (127 μm versus 130 μm reduction) . There are a number of possible reasons for this. The baseline Vas was similar (62 versus 66 letters), but the baseline CRT was significantly higher in our study (471 versus 380 μm) with a higher baseline HbA1c (8.2 versus 7.3), and a greater proportion of eyes having previously had laser (66 versus 54% macular laser, and 39 versus 19% prior PRP). Similarly, the chronicity of DMO was probaly longer, all of which could have affected the outcome [30]. The number of RZB injections given was also lower (six versus nine) relating to real-world variability in follow-up and attendance, as others have noted [33]. This may also have affected the prevalence of ERM and VMA in our cohort, although we found no association between a history of previous laser, the duration of the DMO, nor other factors including HbA1c with the occurrence of ERM or VMA.

We acknowledge that the study has several weaknesses. The cohort size was relatively small and non-controlled. It was also real world, and therefore there were no protocol refractions carried out, although visual acuities were measured in a standardised way using ETDRS letter charts. We did not analyse the number of patient visits and the duration between visits; however, we did not find any difference in the number of injections between the subgroups analysed. We only analysed outcomes at 12 months, and it is possible our results could have been different after the induction phase of four injections or using a different regime. SD OCT is limited in its ability to classify cases of complete vitreoretinal attachment or separation, and therefore in the absence of ultrasound findings we could only grade VMA as present or not. Some of the cases without VMA may have had complete vitreoretinal separation at baseline. Our results therefore need to be interpreted with this important caveat in mind. No correction has been made for multiple testing, and so marginally significant results need to be treated with caution. All eyes were included in the analysis, but we found no significant association between eyes within subjects for most of the endpoints studied. By treating patient as a random effect, the within-patient correlation was calculated and tested using ANOVA. For baseline Va, it was high and significant (0.80). However, for post-treatment Va it drops to 0.38 and for change in Va it drops further to 0.26, and is not significant in either case. For all measurements on CRT, the within-patient correlation is non-significant and can be ignored. Because of the high correlation for baseline Va and, to a certain extent, post-treatment Va, mixed modelling was used to check the conclusions.

The study has several strengths including the prospective data collection in the real-world and hence clinically applicable setting. A standardised SD OCT imaging protocol was followed and the AutoRescan function of the Spectralis used, allowing each follow-up OCT scan to be registered and locked to the baseline scan, thus enabling accurate ERM and VMA progression to be analysed. All included eyes had 12 months of follow-up with standardised injection protocol.

In conclusion, we found that VRIA are related to outcome in patients treated with RZB and should be considered in clinical decision making. ERM was associated with a worsened visual and anatomic response and VMA with an improved anatomical response, particularly when spontaneous VMA release occurred during treatment. The presence and severity of ERM were not affected by RZB treatment, in contrast to the worsening of traction that can occur in eyes treated with anti-VEGFs immediately prior to vitrectomy. Further study of these changes in larger prospective trial datasets is warranted.

## Electronic supplementary material

Below is the link to the electronic supplementary material.

Table S1 (DOCX 13 kb)

Table S2 (DOCX 13 kb)
