# Supplementary material for: Vitreoretinal interface abnormalities in patients treatedwith ranibizumab for diabetic macular oedema
Source: Graefes Arch Clin Exp Ophthalmol. 2016 Dec 12;255(4):733–42. doi: 10.1007/s00417-016-3562-0 (PMC5364245; doi:10.1007/s00417-016-3562-0)
Supplement: Supplementary file 2 — (DOCX 13 kb) [file 417_2016_3562_MOESM2_ESM.docx]

Table S2: Extrafoveal ERM Baseline and at 12 months

|  | | 12 month ERM | | |  |
| --- | --- | --- | --- | --- | --- |
|  |  | No ERM | Linear ERM | Plication | ERM at baseline totals |
| Baseline ERM | No ERM | 35 | 3 | 0 | 38 |
|  | Linear ERM | 3 | 28 | 6 | 37 |
|  | Plication | 0 | 9 | 20 | 29 |
| 12 month ERM totals | | 38 | 40 | 26 | 104 |

P=0.61, Mcnemar’s test

ERM; epiretinal membrane
